# Volumetric growth rates of meningioma and its correlation with histological diagnosis and clinical outcome: a systematic review

**DOI:** 10.1007/s00701-016-3071-2

**Published:** 2017-01-18

**Authors:** Daniel M. Fountain, Wai Cheong Soon, Tomasz Matys, Mathew R. Guilfoyle, Ramez Kirollos, Thomas Santarius

**Affiliations:** 1 Division of Neurosurgery, Department of Clinical Neurosciences, University of Cambridge, Cambridge, UK; 2 Department of Neurosurgery, Cambridge University Hospitals NHS Foundation Trust, Cambridge, UK; 3 University of Edinburgh, Edinburgh, UK; 4 Department of Radiology, Cambridge University Hospitals NHS Foundation Trust, Cambridge, UK

**Keywords:** Meningioma, Volumetric, MRI, Histology, Progression-Free Survival

## Abstract

**Introduction:**

Tumour growth has been used to successfully predict progression-free survival in low-grade glioma. This systematic review sought to establish the evidence base regarding the correlation of volumetric growth rates with histological diagnosis and potential to predict clinical outcome in patients with meningioma.

**Methods:**

This systematic review was conducted according to the PRISMA (Preferred Reporting Items for Systematic Reviews and Meta-Analyses) guidelines. Databases were searched for full text English articles analysing volumetric growth rates in patients with a meningioma.

**Results:**

Four retrospective cohort studies were accepted, demonstrating limited evidence of significantly different tumour doubling rates and shapes of growth curves between benign and atypical meningiomas. Heterogeneity of patient characteristics and timing of volumetric assessment, both pre- and post-operatively, limited pooled analysis of the data. No studies performed statistical analysis to demonstrate the clinical utility of growth rates in predicting clinical outcome.

**Conclusion:**

This systematic review provides limited evidence in support of the use of volumetric growth rates in meningioma to predict histological diagnosis and clinical outcome to guide future monitoring and treatment.

**Electronic supplementary material:**

The online version of this article (doi:10.1007/s00701-016-3071-2) contains supplementary material, which is available to authorized users.

## Introduction

Meningiomas originate from the arachnoid cap cells, which are found in the meninges that surround the brain and spinal cord. Meningiomas are thought to be the most common benign intracranial tumour [20]. The World Health Organisation (WHO) classification system for brain tumours, including meningiomas, was first published in 1979 with the latest edition published in 2016. This and the immediately preceding (2007) WHO classifications do not differ in their grading of meningiomas into benign (grade I), atypical (grade II) and anaplastic (grade III) [13]. A comparative table of histological grading of meningioma is shown in Table 1 [1, 22]. Research has shown that higher-grade tumours are associated with a higher rate of recurrence and a poorer prognosis [24]. Histologically, these tumours have higher mitotic activity and cellular atypia [1]. However, in some cases, benign meningiomas have also been shown to have relatively rapid growth and recurrence after total removal [12, 15].

**Table 1:** Overview of meningioma grading according to the WHO classifications used in the included studies

Classification	I	II	III
1979	Histologic sub-types included meningotheliomatous, fibrous, transitional, psammomatous, angiomatous, haemangioblastic*, haemangiopericytic*, papillary, and anaplastic. Six histologic parameters, each graded 0–3 points, with the overall score below determining the grade of the tumour: 1. Loss of architecture 2. Increased cellularity 3. Nuclear pleomorphism 4. Mitotic figures 5. Focal necroses 6. Brain infiltration (present =3, absent =0)		
	0–2 points	3–6 points	7–11 points
1993	Histologic sub-types added included microcystic, secretory, clear cell, chordoid, lymphoplasmacytic, metaplastic, atypical. Haemangioblastic and haemangiopericytic sub-types removed.		
2000	1. Histological sub-type 2. Lack of anaplastic features	1. Choroid / clear cell histologic sub-type 2. 4–19 mitosis per ten high-power field (HPF) 3. Three or more of 5 features (small cell change, increased cellularity, prominent nucleoli, sheet-like growth, or necrosis)	1. Rhabdoid (added to classification) / papillary histologic sub-type 2. Histological picture of frank malignancy resembling carcinoma / melanoma / high grade sarcoma 3. >20 mitosis per ten HPF
2007		Fourth criteria added: 4. Brain infiltrative and otherwise benign meningiomas	

^*^ These tumours were excluded in the Jaaskelainen series as they were considered to be different tumours. They were subsequently removed in the 1993 classification as shown in the table

In addition to histological diagnosis, growth rate has been found to serve as significant prognostic factor in low grade glioma [19]. We hypothesised that this could be similarly useful in the longitudinal management of patients with meningiomas. We thus sought to design a systematic review to ascertain the current evidence base for the prognostic value of spontaneous volumetric growth of meningiomas as seen on MRI. We also aimed to establish the relationship between the rate of meningioma growth and the underlying WHO meningioma histological grades and identify whether the combination of these two parameters has been used to predict recurrence, overall survival and anaplastic transformation.

## Methods

This systematic review was conducted according to PRISMA guidelines and has been registered with the PROSPERO international prospective register of systematic reviews (registration number CRD42016027746).

### Search strategy

A systematic search of keywords in Table 2 was performed independently by two authors (DMF and WCS) of MEDLINE Complete, EMBASE 1974 to 2015 via Ovid and the Cochrane Central Register of Controlled Trials (CENTRAL) via the Cochrane Library on the 1st January 2016. A record of our MEDLINE search is provided in the Supplementary Material (Table S1).

**Table 2:** Terms used in the systematic search. MeSH terms are shaded in grey

Diagnosis	Modality	Imaging Measurement	Outcome Measure
Meningioma	Magnetic Resonance Imaging	Grow*	Disease Progression
Meningioma*	Tomography, X-Ray Computed	Morpholog*	Disease-Free Survival
	MRI	Volum*	Prognosis
	MR	Imaging feature*	Survival
	Magnetic Resonance Imaging		Recurrence
	CT		Neoplasm Grading
	Computed Tomography		Histology
			Histolog*
			Histopatholog*
			Prognosis
			Survival
			Recurrence
			Anaplastic Transformation

Initially titles and abstracts were screened for relevant papers by two authors independently (DMF and WCS). Decisions were blinded and, where disagreements occurred, both authors discussed the disparities and resolved them throughout the selection process. The bibliographies of accepted papers were also examined for additional articles not identified in the systematic search. The inclusion criteria, designed using the PICO (Patient and Problem, Intervention, Comparators, Outcomes) process, were as follows:

- *Study design*: Randomised controlled trials, controlled clinical trials, prospective and retrospective observational studies were included conditional upon a sample size > 10 patients from the population of interest. We excluded studies with smaller sample sizes, such as case reports and case series, on the grounds of a potential publication bias risk. Only full articles in English language were included, with articles in other languages, conference abstracts and grey literature excluded.
- *Population*: All studies including human subjects with a meningioma.
- *Intervention*: Patients underwent imaging with accompanied volumetric growth analysis.
- *Outcome*: All studies investigating correlation rates of growth analysis with histological diagnosis, progression-free and overall survival, recurrence rate and rates of anaplastic transformation were included.

Data extraction was also performed by two authors (DMF and WCS) to ensure reliability. Forward and backward searching of included studies provided one further study not identified in the database search.

## Results

**Fig. 1:**
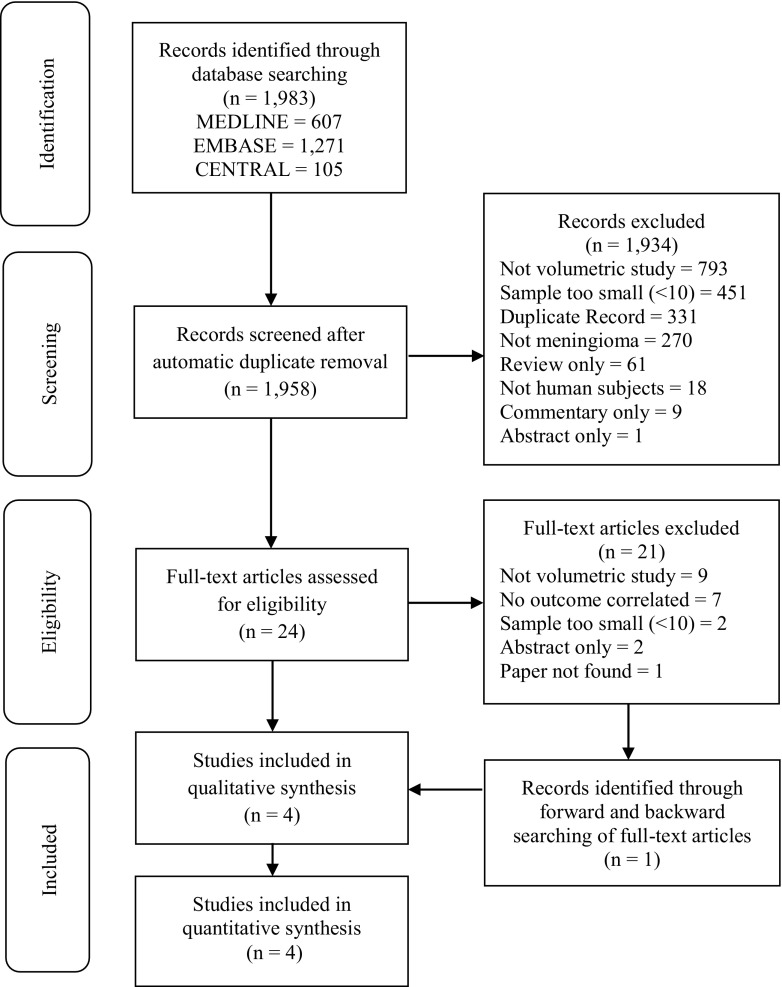
Flow Diagram of Systematic Search Process

Our search strategy identified a total of 1,983 articles for screening. After excluding duplicates and irrelevant studies, we obtained 24 full articles for further assessment of eligibility. Of these, 21 were excluded mainly because the studies did not include volumetric growth rates or the volumetric growth rates were not directly correlated with the outcomes (Fig. 1). Four retrospective cohort studies with a total of 151 patients met the inclusion and exclusion criteria for the systematic review. As such, they were all classified as evidence level 2b [8]. Table 3 shows a summary of the four studies that have been included in the analysis.

**Table 3:** Summary of Accepted Studies M = Male, F = Female. Inflection point refers to point on a curve at which the curve changes from concave convex or vice versa

Study	Population (n, sex, age, histology)	WHO Classification	Method Used for Volumetric Calculation	Doubling time (mean days)	Outcome measure (histology, recurrence)	Findings
Jääskeläinen et al. 1985[10]	43 (14 M, 29 F), mean age 47 years (range 16–65) Benign – 24 Atypical – 12 Malignant - 7	Corresponding to 1979 grading	Post-operative recurrence after radical removal measured via CT - area of tumour measured using computed planimeter and multiplied by slice thickness.	Grade I - 415 (range 138–1,045) Grade II - 178 (range 34–551) Grade III - 205 (range 30–472)	Compared to initial resection, 4 benign tumours became atypical and 4 anaplastic tumours became malignant.	Highly significant difference between grade I and grade II-III (Kruskal-Wallis one-way ANOVA *p* < 0.001), but not between grades II and III. Correlation between the mitotic index and the doubling time highly significant (Spearman rank correlation coefficient r = − 0.519, *p* < 0.001). Atypical meningiomas tend to grow quasi-exponentially. 23/31 asymptomatic meningiomas had already passed the inflection point prior to diagnosis.
Nakamura et al. 2005[13]	36 (10 M, 26 F) Grade I – 33 (7 M, 26 F. Mean age 53.14, range 33–79) Grade II - 3 (3 M. Mean age 45.5, range 37–60)	2000*	Post-operative with remnant tumours determined using areas measured on MRI / CT using NIH image programme (Scion image J) multiplied by slice thickness. Each volume measured three times, and the mean value calculated.	Grade I = 1908 (median) Grade II = 204 (median)	Mean absolute annual growth rate: Grade I =1.51 cm^3^/year Grade II = 23.3 cm^3^/year	Growth rates may wary widely even among Grade I meningiomas. Growth rates for younger patients and Grade II meningiomas are significantly higher. Hypo or iso-intense T2 signals and/or presence of calcification on MRI correlate with a lower growth rate.
Nakasu et al. 2005[15]	20 (5 M, 15 F), median age 55 Grade I – 9 Grade II – 4 Histology not available – 7	2000	Pre-operative volumes determined using areas measured on MRI / CT using National Institutes of Health (Bethesda, MD) Image 1.62 multiplied by slice thickness. Each volume measured three times, and the mean value calculated.	Benign – 2,198 Atypical – 322 Incidental – 3,223	Grade I – 8 regrowth, 1 recurrence Grade II – 1 regrowth, 3 recurrence	Growth curves of atypical meningiomas fitted better to the exponential curve (R > 0.94). Although post-resection re-growth was faster than pre-resection in three patients, this was not statistically significant (Wilcoxon matched-pairs test, *p* = 0.14). Meningiomas without calcification are likely to grow exponentially.
Nakasu et al. 2011[16]	52 (13 M, 39 F), median age 57.6 31 – Asymptomatic 21 – Post-operative (Grade I – 15, Grade II – 6)	2007	Asymptomatic and post-operative volumes determined using areas measured on MRI / CT using National Institutes of Health (Bethesda, MD) Image 1.62 multiplied by slice thickness. Each volume measured three times, and the mean value calculated.	N/A	One benign tumour enlarged about 8 times during the first 79 months.	Atypical meningiomas unlikely to pass point of tumour growth deceleration compared with benign meningiomas (log-rank test, *p* = 0.04). No difference of patient’s age at inflection point between symptomatic and asymptomatic meningiomas. Benign meningiomas may pass the inflection point and approach their plateau volume in the long run, while atypical meningiomas are not likely to do so.

*This is assumed as the text does not explicitly state this and no classification is cited

### Volumetric calculation methods

The methods used for volumetric measurements varied between studies. Jääskeläinen et al. [10] used computed planimeter to measure the volumetric growth rates on CT images whereas Nakasu et al. [16, 17] performed volumetric measurements on CT and MRI images using the National Institutes of Health (Bethesda, MD) Image 1.62 software. Nakamura et al. determined the mean volume from three volumetric measurements using the National Institutes of Health Scion Image J programme [14].

### Histology grades

Only Nakasu et al. 2011 [17] cited the revised 2007 World Health Organisation (WHO) classification system. It is assumed authors used the 2007 classification system when assessing the histology of the tumours although the patients included in their study had resection of their meningiomas from 1980 to 2004. The rest of the studies correlated the volumetric growth rates with the pre-2007 WHO histology grading.

Of the available histological grades, there were 81 grade I or benign meningiomas, 21 grade II or atypical meningiomas and 7 grade III or malignant meningiomas. Histology was not available in 38 cases as the patients were asymptomatic and did not undergo biopsy or resection of their tumours. Two studies undertook growth rate analysis post-operatively; whilst Jääskeläinen et al. reported growth rates following recurrence after radical removal [10], Nakamura reported growth rates following identification of remnant tumour post-operatively [14]. Whilst Nakasu et al. 2005 reported pre-operative volumes [16], Nakasu et al. 2011 analysed a combination of asymptomatic and post-operative meningioma, the latter either as a remnant or a recurrence [17]. Jääskeläinen et al. reported that four benign meningiomas transformed into atypical meningiomas and four atypical tumours transformed into malignant meningiomas [10]. In the 2005 study by Nakasu et al., the authors described eight out of nine Grade I and one out of four Grade II meningiomas presenting as regrowth, the remaining presenting as recurrence [16].

### Mean growth rates and tumour doubling time

The mean growth rates (cm^3^/year) were only specified in Nakamura et al., reporting significantly higher rates in grade II meningioma relative to grade I [14]. The tumour doubling time in days were specified in three of the four studies. Jääskeläinen identified a significantly shorter tumour doubling time between grade I and grade II or III tumours,[10] which was consistent with findings in Nakamura reporting significantly higher growth rates in grade II meningioma [14].

The pattern of growth also showed evidence of utility in predicting histological grade. It was reported that atypical meningiomas tend to grow quasi-exponentially and a majority of benign or asymptomatic meningiomas had already passed the inflection point prior to diagnosis [10, 17]. The majority of grade I meningiomas begun to slow their growth before the age of 80 years when compared with the growth of atypical meningiomas which did not significantly show deceleration (*p* = 0.04) [17]. Using regression analysis, the authors showed that the growth curves of meningiomas fitted the logistic and sigmoid Gompertzian curves. In addition, the authors also demonstrated that the growth rates of benign meningiomas may change in the long term and it is vital to apply the correct growth model to predict tumour growth and guide the decision-making process.

### Other findings

Nakamura et al. highlighted that the growth rates of benign meningiomas vary widely even among grade I meningiomas [14]. Factors associated with an increased volumetric growth rate include younger age at diagnosis [14], lack of calcification on radiological scans [16] and the presence of hyper-intense T2 signals on MRI [14].

Table 4 presents twelve studies that did not meet the inclusion or exclusion criteria but we felt that the findings of the papers should be reviewed as volumetric growth rates were performed in these studies. The demographic data could not be summarised as they are not uniformly available. Two out of twelve studies investigated volumetric growth rates in neurofibromatosis type 2-associated meningiomas [3, 5]. Evers et al. showed that in patients with NF2-associated meningiomas, tumours in the skull base have higher absolute growth rates when compared to convexity and ‘other’ meningiomas [5]. In the study by Dirks et al., the authors found that younger age at diagnosis (*p* = 0.01) and female gender (*p* = 0.05) significantly contributed to increased volumetric growth rates [3].

**Table 4:** Summary of Studies Evaluating Volumetric Growth Rates M = Male, F = Female. M = Male, F = Female

Study	Population (n)	Method Used for Volumetric Calculation	Mean tumour growth rates (cm3)	Doubling time	Outcome measure (histology, recurrence)	Findings
Nakamura et al. 2003 [13]	41 patients (4 M, 37 F) Mean age 60.9 years (range 33–84) Mean follow-up 43 months (range 6–105)	Post-contrast CT or MRI scans with 3–7 mm thick slices. Volume calculated using the NIH image program (Scion Image J).	Annual growth rate absolute 0.03–2.62 cm^3^, relative 0.48–72.8% (mean 14.6%).	Mean 21.6 years (1.27 to 143.5 years)	Growth rates correlated with patient age, radiological features including presence of calcification and presence of hyperintensity, and need for surgical intervention.	Tumour size moderate positive correlation with absolute annual growth rate, moderate negative correlation with relative annual growth rate, and no correlation with tumour doubling time. Age was the only predictive factor that correlates with growth. Presence of calcification or hypointense / isointense T2 signals associated with lower tumour growth rate and a longer tumour doubling time.
Evers et al. 2015 [5]	210 NF2- associated meningiomas, 21 (8 M, 13 F) mean age 28.48 Mean follow-up = 5.5 years Tumour location:Skull base - 85 Convexity - 31 Other location −91	The tumours are manually marked on MRI scans using Brainlab software iPlan Cranial 2.6.1 on each MRI scan. Localisation of the tumours were done by experienced neurosurgeon or radiologist.	Skull base - 0.21 Convexity - 0.09 Other - 0.08	N/A	Growth rates correlated with tumour locations (skull base, convexity and ‘other’ [parasagittal, falx, tentorial, intraventricular, intraorbital] meningiomas)	Skull base tumours have a higher absolute growth rates compared to convexity and ‘other’ meningiomas
Oya et al. 2011 [18]	273 meningiomas (53 M, 220 F) in 244 patients.Mean age 60.5 years	The contour of each tumour were manually traced on each slice image using freehand tools on ImageJ version 1.43. The volume of the tumours were calculated by multiplying tumour areas with the slice thickness of the image.	N/A	N/A	Growth rates correlated with the age of patients and radiological features such as presence of calcification, peritumoural oedema, T2 Hyperintensity and initial tumour diameter	Absence of calcification, presence of T2 hyperintensity, age 60 and younger, initial diameter greater than 25 mm, and oedema associated with higher annual growth rates. Volumetric growth rates observed in 74% of cases (114/154)
Hashiba et al. 2009 [6]	70 (9 M, 61 F), 7 excluded. Mean age - 61.6. Mean follow-up 39.3 months. Growth group = 44. No growth = 26	Using the Scion Image for Windows (Scion Corp), the enhanced area of the tumours were calculated by manually tracing the boundaries of each tumour and multiplying the sum enhanced area of tumours with the slice thickness of the MRI images.	Linear group - 14.87%. Exponential - 25.5%. Other’- 15.53%	Mean tumour doubling time = 2808 days (range 390 to 7,020)	Growth rates correlated with patient demographics such as age and gender and radiological features such as initial tumour volume, location, T2 signal intensity and peritumoral oedema.	Growth <15% considered measurement error Presence of calcification distinguished tumour growth group from non-growth group There were no significant difference in gender, age, initial tumour volume, tumour location, peritumoral oedema and T2 signal hyperintensity when comparing growth group with non-growth group
Firsching et al. 1990 [6]	17 (3 M, 14 F), Age range 46–83. Histology result available in one case but grade not reported.	Tumour areas were measured by planimeter and volumes were calculated by multiple scale-adjusted tumour areas in squared millimeters with the distance between adjacent CT or MRI scans.	Annual growth rates ranged from 0.5% to 21%, median of 3.6%	N/A	Growth rates correlated with patients’ age, duration of follow-up and initial tumour volume	Tumour growth rate not related to age and there was no relationship between growth rates and initial tumour volume or follow-up period
Chang et al. 2012 [2]	31 patients (mean age 62.7) Median follow-up - 4.1 years	Volumetric measurements were performed on T1 post-contrast axial images using the semi-automated user-assisted tumour volume measuring feature of Vitrea Medical Imaging software. Tumour volumes were converted to diameter using the formula (Diameter = (2 x Volume)/3	Perimetric method - mean growth rate = 15.2% [0.7 mm/year] (significant growth in 19 patients) Diameter method - mean growth rate = 5.6% [0.8 mm/year] (significant growth in 12 patients)	Perimetric method = 4891 days Diametric method = 6533.5 days	Growth rates using perimeter method and diametric method were directly compared. Growth rates were also correlated with patients’ age and radiological features such as tumour location, initial tumour volume, peritumoral oedema and length of follow-up	Measurement using perimeter method showed a significantly higher annual growth rate and lower tumour doubling time when compared to the diameter method (p < 0.01). Age at diagnosis, tumour location, initial tumour volume, length of follow-up, oedema, irregular margins did not distinguish growing from non-growing tumours.
Hashimoto et al. 2012 [7]	110 patients (17 M, 93 F), mean age =66.8 mean follow-up = 46.9 years 113 Incidentally-discovered meningiomas 38 Skull base, 75 non-skull base meningiomas	Using the Scion Image for Windows (Scion Corp), the enhanced area of the tumours were calculated by manually tracing the boundaries of each tumour and multiplying the sum enhanced area of tumours with the slice thickness of the MRI images.	15/38 SB showed growth (1.2 cm3/year) - mean growth 25.56% 56/78 Non-SB showed growth (1.15 cm3/year) - mean growth 94.83%	SB - 4,824 days Non- SB - 3,334.5 days	Growth rates in skull base versus non-skull base were directly compared and also correlated with MIB-1 index	Tumour doubling time was significantly lower (p = 0.008) and percentage of growth (p = 0.002) was significantly higher in non-skull base meningiomas. MIB-1 index was significantly lower in skull base tumours (p = 0.013). Mean MIB-1 index was significantly higher in male with non-skull base tumours (p = 0.021)
Yoneoka et al. 2000 [25]	37 patients (5 M,32 F)	Volumes were calculated from CT or MRI scan of 5 mm thickness by using the US National Institute of Health (NIH) image programme. The annual growth rates of the tumours based on the difference in tumour volume between the latest and initial scans divided by the time-interval of the follow-up period	9/37 patients showed growth (>1 cm3/year) Mean tumour growth in these patients [5.3 +/− 2.1 cm3/year]	N/A	Growth rates correlated with demographics such as age and gender and pre-operative radiological features such as tumour location, initial tumour volume, mass effect and calcification.	Tumour growth significantly higher in younger patients (p = 0.042) and in patients with higher initial tumour volume (p = 0.042). No significant difference between volumetric tumour growth and gender or length of follow-up.
Ide et al. 1995 [9]	12 patients (4 M, 8 F), mean age = 60.7 years Grade I Meningiomas	Using the planimeter (Ushikata X-plan 360d; Ushikata Inc., Tokyo), the tumour volume was measured by multiplying the sum area of tumours with slice thickness (5 mm).	41.425 /12 = 3.452	3084.08 days (range 197–7943)	Growth rates correlated with proliferating cell nuclear antigen (PCNA) immunostaining	There is a significant inverse relationship between PCNA indexes and tumour doubling times (p = 0.003). Meningiomas with >1% of PCNA staining indexes have shorter tumour doubling times of <5 years.
Zeidman et al. 2008 [26]	21 patients (7 M, 14 F), mean age = 61.0 years Mean follow-up = 3.64 years	Tumour volumes were calculated through serial MRI scans using ellipsoid formula = (AxBxC)/2 where a, b and c represent the three perpendicular axes of each tumour.	Relative growth rate =5.82%/year Relative growth diameter = 2.00%/year	N/A	Growth rates correlated with demographics such as age and gender, radiological characteristics such as tumour location, calcification, T2-signal intensity on MRI, dural tail, mass effect and midline shift)	There was a significant difference between the mean relative volumetric and planimeter growth rate (p < 0.0001). No significant association between tumour location, age, gender, radiological characteristics and volumetric growth rates.
Dirks et al. 2012 [3]	NF2- associated meningiomas. 13 patients (139 meningiomas),unclear about exact demographics	The tumour volumes were calculated using the ellipsoid formula (AxBxC)/2 on serial post-contrast T1-weighted MRI imaging.	Mean = 0.4	N/A	Correlated with demographics such as age, gender, Karnofsky Perfomance Scale (KPS) and family history of NF2 and radiological findings such as T2 signal intensity, tumour location, presence of peritumoral cyst, peritumoral oedema and duration of follow-up	A younger age at onset (p = 0.01) and female gender (p = 0.05) were associated with an increased volumetric growth rate of meningiomas. NF2-associated tumours demonstrate a saltatory growth pattern.
Jung et al. 2000 [11]	38 Subtotally resected petroclival meningiomas. Male : Female ratio = 5.33. Median age = 47.6 [15–63 years]	The tumour volumes were calculated using the ellipsoid formula (A × B × C)/2 on MRI / CT scans.	Mean = 4.94 cm3/year	2906 days (range 114–8918)	Growth rates correlated with patients’ age, gender, onset of menopause, tumour size, duration of symptoms, Karnofsky Performance Scale (KPS), post-operative neurological deficits, extent of removal, lobular growth pattern, and involvement of vertebrobasilar artery.	Tumour growth rate is significantly slower in older patients (>50 y). Tumour progression rate in patients with subtotal resection is 42%. Reoperation and/or radiotherapy produced good results in patients with progressive disease.

Two studies demonstrated that computer-aided volumetric growth analysis is significantly more superior to traditional methods in assessing tumour growth [2, 27]. There were eight different methods used for performing volumetric growth rates in these studies as demonstrated in Table 4. Two studies used the ellipsoid formula (a × b × c)/2 whereby a, b and c represent the maximum width, length and height respectively to calculate the tumour volume [3, 27]. Information on histological grading of the meningiomas was only reported in one study [9]. The mean relative growth rates and tumour doubling time are not comparable due to the heterogeneity of the reported data.

## Discussion

The majority of meningiomas are generally benign and slow-growing tumours. In clinical practise, tumour growth together with the presence of symptoms are the main determining factors of the frequency of follow-up, both pre- and post-operatively, and help guide the clinical management decision-making process. Nevertheless, there remains limited information in the literature regarding the growth rates of meningioma. A range of imaging methods is available for assessing and monitoring growth and disease progression [2, 5, 6, 18]. Volumetric measurements have been found to be superior to traditional planimetric methods in assessing tumour growth kinetics. This has been proposed to be due to tumours being three-dimensional structures that may grow in various directions and not just in the planimetric axes [2, 23].

When reviewing evidence on radiological follow-up of low-grade gliomas, Pallud et al. found that there remains a question as to whether growth should be monitored over time in diameter or in volume [19]. This is due to the fact that the tumour volumes do not usually increase linearly when compared with tumour diameter. The authors proposed that the linearity of diameter growth curves will allow the analysis of biological aggressiveness to be more straightforward. The tumour volumes can be converted into diameter using the formula Mean Tumour Diameter (MTD) = (2 × Volume)^1/3^. The MTD can then be used to calculate the velocity of diametric expansion (VDE) by plotting the MTD and performing a linear regression of VDE over time [19]. The authors concluded that spontaneous VDE carries a strong prognostic significance with regards to progression-free and overall survivals in patient with low-grade glioma. There is currently no evidence if this metric is applicable to meningiomas.

According to the CBTRUS statistical report, the incidence of meningioma increases with age but there is no specific mention as to whether older age at initial diagnosis correlates with higher-grade meningioma [4]. However, Park JS et al. has shown that the combined incidence of WHO grade II and III increases as age advances [21]. Several studies have demonstrated that a higher tumour volume at initial diagnosis is significantly associated with a higher rate of tumour recurrence [2, 26]. However, little is known regarding the volume of the tumour and the probability that the tumour is of a higher grade. Using diffusion-tensor MR imaging, Wang et al. showed that there is significant difference in tumour volume between atypical and benign meningioma. Atypical meningioma tended to be larger when compared to benign meningioma [25].

Our review has indicated that the growth rates of meningiomas may evolve over time and vary even among grade I meningiomas [14, 15]. Jääskeläinen et al. and Nakamura et al. showed that higher-grade tumours are significantly more likely to have higher growth rates and shorter tumour doubling times [10]. However, these studies have correlated the volumetric growth rates with the old WHO classification of meningiomas. None of the studies attempted to correlate the growth rates of meningiomas with the revised 2007 WHO classification of meningiomas. Moreover, the findings of these studies are limited by the small number of patients and their retrospective nature. A reliable method to evaluate spontaneous growth rates of meningiomas on MRI should be utilised to determine its usefulness in determining the frequency of follow-ups, determine progression-free survival, treatment efficacy and, possibly, the probability of recurrence or regrowth after resection.

With regards to the growth rate of non-operated tumours, Zeidman et al. showed that there was no significant association between age, gender, or radiological characteristics and volumetric growth [27]. These results need to be considered in the context of the cohort size of only 21 patients. In a multi-variate analysis of a slightly larger series (*N* = 37) by Yoneoka et al., tumour growth was significantly higher in younger patients and in patients with a higher volume of tumour at initial diagnosis [26]. When looking at the natural history of 273 intracranial meningiomas, Oya et al. demonstrated that volumetric growth rate was seen in 74% of cases. Factors such as male sex, initial tumour diameter greater than 25 mm, MR imaging T2 hyperintensity, presence of symptoms and oedema are significantly associated with higher annual growth rate [18]. Interestingly, Evers et al. demonstrated that in patients with neurofibromatosis type-2 meningiomas, skull base tumours have higher growth rates compared with convexity and other locations whereas Hashimoto et al. demonstrated that in non-NF2 related meningiomas, tumour doubling-time was significantly lower in non-skull base tumours [5, 7].

## Limitations

The review was limited by the level of evidence in accepted studies, with four retrospective cohort studies accepted. Heterogeneity of patient demographics and the timing of volume measurement prevented pooled analysis of the data. None of the studies undertook statistical analysis to investigate the potential for volumetric growth rates to act as a predictor of histological diagnosis or clinical outcome, restricting to demonstrating significant differences between histological diagnoses.

## Conclusion

Tumour volumetric growth rates were hypothesised to be a potentially useful indicator of histological diagnosis and prognosis in patients with meningioma. This systematic review of volumetric growth rates in patients with meningioma identified four retrospective cohort studies providing limited evidence in favour of the correlation of volumetric growth rates with histological diagnosis. None of the accepted studies extended this analysis to evaluate the potential to predict clinical outcome. This study highlighted a knowledge gap that is ready to be addressed as digital acquisition and storage of radiological imaging has been widely available for over a decade in most neurosurgical centres.

## Electronic supplementary material

Below is the link to the electronic supplementary material.

ESM 1 (DOCX 13 kb)

## References

[1] Backer-Grøndahl T, Moen BH, Torp SH (2012). The histopathological spectrum of human meningiomas. Int J Clin Exp Pathol.

[2] Chang V, Narang J, Schultz L, Issawi A, Jain R, Rock J, Rosenblum M (2012). Computer-aided volumetric analysis as a sensitive tool for the management of incidental meningiomas. Acta Neurochir (Wien).

[3] Dirks MS, Butman JA, Kim HJ, Wu T, Morgan K, Tran AP, Lonser RR, Asthagiri AR (2012). Long-term natural history of neurofibromatosis Type 2-associated intracranial tumors. J Neurosurg.

[4] Dolecek TA, Propp JM, Stroup NE, Kruchko C (2012). CBTRUS statistical report: primary brain and central nervous system tumors diagnosed in the United States in 2005–2009. Neuro-Oncol.

[5] Evers S, Verbaan D, Sanchez E, Peerdeman S (2015). 3D Volumetric Measurement of Neurofibromatosis Type 2-Associated Meningiomas: Association Between Tumor Location and Growth Rate. World Neurosurg.

[6] Hashiba T, Hashimoto N, Izumoto S, Suzuki T, Kagawa N, Maruno M, Kato A, Yoshimine T (2009). Serial volumetric assessment of the natural history and growth pattern of incidentally discovered meningiomas. J Neurosurg.

[7] Hashimoto N, Rabo CS, Okita Y (2012). Slower growth of skull base meningiomas compared with non-skull base meningiomas based on volumetric and biological studies. J Neurosurg.

[8] Howick J (2009) Oxford Centre for Evidence-based Medicine – Levels of Evidence. University of Oxford, Centre for Evidence-Based Medicine

[9] Ide M, Jimbo M, Yamamoto M, Umebara Y, Hagiwara S, Kubo O (1995). Growth rate of intracranial meningioma: tumor doubling time and proliferating cell nuclear antigen staining index. Neurol Med Chir (Tokyo).

[10] Jääskeläinen J, Haltia M, Laasonen E, Wahlström T, Valtonen S (1985). The growth rate of intracranial meningiomas and its relation to histology. An analysis of 43 patients. Surg Neurol.

[11] Jung HW, Yoo H, Paek SH, Choi KS (2000). Long-term outcome and growth rate of subtotally resected petroclival meningiomas: experience with 38 cases. Neurosurgery.

[12] Kudoh C, Sugiura K, Yoshimizu N, Detta A (1995). Rapidly growing histologically benign meningiomas: cell kinetic and deoxyribonucleic acid ploidy features: report of three cases. Neurosurgery.

[13] Louis DN, Perry A, Reifenberger G, von Deimling A, Figarella-Branger D, Cavenee WK, Ohgaki H, Wiestler OD, Kleihues P, Ellison DW (2016). The 2016 World Health Organization Classification of Tumors of the Central Nervous System: a summary. Acta Neuropathol (Berl).

[14] Nakamura M, Roser F, Michel J, Jacobs C, Samii M (2005). Volumetric analysis of the growth rate of incompletely resected intracranial meningiomas. Zentralblatt Für Neurochir.

[15] Nakasu S, Fukami T, Jito J, Nozaki K (2009). Recurrence and regrowth of benign meningiomas. Brain Tumor Pathol.

[16] Nakasu S, Fukami T, Nakajima M, Watanabe K, Ichikawa M, Matsuda M (2005). Growth pattern changes of meningiomas: long-term analysis. Neurosurgery.

[17] Nakasu S, Nakasu Y, Fukami T, Jito J, Nozaki K (2011). Growth curve analysis of asymptomatic and symptomatic meningiomas. J Neurooncol.

[18] Oya S, Kim S-H, Sade B, Lee JH (2011). The natural history of intracranial meningiomas. J Neurosurg.

[19] Pallud J, Taillandier L, Capelle L, Fontaine D, Peyre M, Ducray F, Duffau H, Mandonnet E (2012). Quantitative morphological magnetic resonance imaging follow-up of low-grade glioma: a plea for systematic measurement of growth rates. Neurosurgery.

[20] Park BJ, Kim HK, Sade B, Lee JH, Lee JH (2009). Epidemiology. Meningiomas.

[21] Park J-S, Sade B, Oya S, Kim CG, Lee JH (2014). The influence of age on the histological grading of meningiomas. Neurosurg Rev.

[22] Scheithauer BW (2009). Development of the WHO classification of tumors of the central nervous system: a historical perspective. Brain Pathol Zurich Switz.

[23] Vakilian S, Souhami L, Melançon D, Zeitouni A (2012). Volumetric Measurement of Vestibular Schwannoma Tumour Growth Following Partial Resection: Predictors for Recurrence. J Neurol Surg Part B Skull Base.

[24] Violaris K, Katsarides V, Karakyriou M, Sakellariou P (2013). Surgical Outcome of Treating Grades II and III Meningiomas: A Report of 32 Cases. Neurosci J.

[25] Wang S, Kim S, Zhang Y, Wang L, Lee EB, Syre P, Poptani H, Melhem ER, Lee JYK (2012). Determination of grade and subtype of meningiomas by using histogram analysis of diffusion-tensor imaging metrics. Radiology.

[26] Yoneoka Y, Fujii Y, Tanaka R (2000). Growth of Incidental Meningiomas. Acta Neurochir (Wien).

[27] Zeidman LA, Ankenbrandt WJ, Du H, Paleologos N, Vick NA (2008). Growth rate of non-operated meningiomas. J Neurol.

