# Supplementary material for: Volumetric growth rates of meningioma and its correlation with histological diagnosis and clinical outcome: a systematic review
Source: Acta Neurochir (Wien). 2017 Jan 18;159(3):435–45. doi: 10.1007/s00701-016-3071-2 (PMC5306174; doi:10.1007/s00701-016-3071-2)
Supplement: Supplementary file 1 — (DOCX 13 kb) [file 701_2016_3071_MOESM1_ESM.docx]

**Supplementary Material**

*Table S1 – MEDLINE via EBSCOhost Search Strategy – Conducted 1^st^ January 2016*

| **Search ID#** | **Search Terms** | **Search Options** | **Last Run Via** | **Results** |
| --- | --- | --- | --- | --- |
| S12 | S3 AND S7 AND S8 AND S11 | Search modes - Boolean/Phrase | Interface - EBSCOhost Research Databases  Search Screen - Advanced Search  Database - MEDLINE Complete | 607 |
| S11 | S9 OR S10 | Search modes - Boolean/Phrase | Interface - EBSCOhost Research Databases  Search Screen - Advanced Search  Database - MEDLINE Complete | 2,427,748 |
| S10 | Histolog* OR Histopatholog* OR Prognosis OR Survival OR Recurrence OR Anaplastic Transformation | Search modes - Boolean/Phrase | Interface - EBSCOhost Research Databases  Search Screen - Advanced Search  Database - MEDLINE Complete | 2,354,520 |
| S9 | (MH "Disease Progression") OR (MH "Disease-Free Survival") OR (MH "Prognosis") OR (MH "Survival") OR (MH "Recurrence") OR (MH "Neoplasm Grading") OR (MH "Histology") | Search modes - Boolean/Phrase | Interface - EBSCOhost Research Databases  Search Screen - Advanced Search  Database - MEDLINE Complete | 660,834 |
| S8 | Grow* OR Morpholog* OR Volum* OR Imaging Feature* | Search modes - Boolean/Phrase | Interface - EBSCOhost Research Databases  Search Screen - Advanced Search  Database - MEDLINE Complete | 2,672,343 |
| S7 | S4 OR S5 OR S6 | Search modes - Boolean/Phrase | Interface - EBSCOhost Research Databases  Search Screen - Advanced Search  Database - MEDLINE Complete | 1,175,966 |
| S6 | Computed Tomography | Search modes - Boolean/Phrase | Interface - EBSCOhost Research Databases  Search Screen - Advanced Search  Database - MEDLINE Complete | 172,793 |
| S5 | MRI OR MR OR Magnetic Resonance Imaging OR CT | Search modes - Boolean/Phrase | Interface - EBSCOhost Research Databases  Search Screen - Advanced Search  Database - MEDLINE Complete | 980,617 |
| S4 | (MH "Magnetic Resonance Imaging") OR (MH "Tomography, X-Ray Computed") | Search modes - Boolean/Phrase | Interface - EBSCOhost Research Databases  Search Screen - Advanced Search  Database - MEDLINE Complete | 548,574 |
| S3 | S1 OR S2 | Search modes - Boolean/Phrase | Interface - EBSCOhost Research Databases  Search Screen - Advanced Search  Database - MEDLINE Complete | 21,351 |
| S2 | Meningioma* | Search modes - Boolean/Phrase | Interface - EBSCOhost Research Databases  Search Screen - Advanced Search  Database - MEDLINE Complete | 21,351 |
| S1 | (MH "Meningioma") | Search modes - Boolean/Phrase | Interface - EBSCOhost Research Databases  Search Screen - Advanced Search  Database - MEDLINE Complete | 16,515 |
